# Safe, Simple, and Rapid: A Minimally Invasive Coding Technique for Identifying Individuals of the Endangered Mongolian Racerunner (*Eremias argus*)

**DOI:** 10.1002/ece3.73438

**Published:** 2026-04-08

**Authors:** Jae Young Song, Min Ho Chang, Seok Beom Kim, Kyo Soung Koo

**Affiliations:** ^1^ Korea National Park Research Institute Wonju South Korea; ^2^ National Institute of Ecology Seocheon South Korea; ^3^ Korea National Park Service Wonju South Korea; ^4^ Korean Environmental Geography Institute Sejong South Korea

**Keywords:** dorsal spot pattern, identification accuracy, non‐invasive methods, population monitoring, wildlife conservation

## Abstract

Individual identification is essential for wildlife population research but commonly relies on invasive methods that may negatively affect animal welfare. This study proposes a safe, simple, and rapid non‐invasive identification method for the endangered Mongolian racerunner (*Eremias argus*) based on coding dorsal spot patterns. We documented 175 individuals using photographic data and assigned unique identification codes derived from two central longitudinal spot lines and overlapping spots, which capture individual‐specific traits. No individuals were found to share identical codes during identification. Recaptured individuals showed stable spot patterns without any code changes, confirming the method's reliability for long‐term monitoring. This approach requires only standard cameras or smartphones and is thus practical for field surveys and citizen science initiatives. The proposed method minimizes impacts on target species while maintaining high accuracy, offering a valuable alternative for ethical conservation and population studies.

## Graphical abstract

The second figure clearly illustrates the key coding method proposed in this paper.

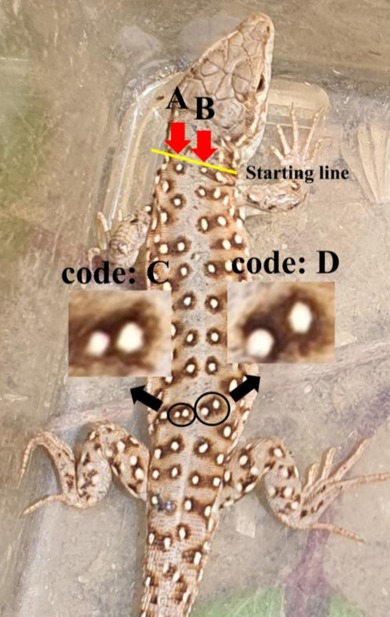

## 1 Introduction

Accurate individual identification constitutes a fundamental component of wildlife population research (Halliday 1996; Silvy et al. 2012). Such individual identification serves as a critical foundation not only for ensuring data reliability, but also for facilitating long‐term ecological monitoring and the development of effective conservation strategies (Pereira et al. 2022; Ellis‐Soto et al. 2025). Commonly employed techniques for individual identification in animals include tissue clipping, microchip implantation, tagging, use of fluorescent markers, and pattern‐based recognition (Camper and Dixon 1988; Ferner 2007; Doody et al. 2009; Furman et al. 2011; Silvy et al. 2012; Zemanova et al. 2025). In lizard species with well‐developed limbs, toe clipping provides a straightforward and cost‐efficient means of individual recognition (Middelburg and Strijbosch 1988). However, this method is not without drawbacks, as toe removal can impair locomotor and reduce survival rates (Halliday 1996; Parris and McCarthy 2001; Zemanova et al. 2025). Moreover, natural toe loss or subsequent regeneration may lead to misidentification of individuals (Middelburg and Strijbosch 1988; Hudson 1996; Vervust et al. 2009), thereby compromising the reliability of research outcomes (McCarthy and Parris 2004; Bloch and Irschick 2005). Conversely, radio telemetry employing externally attached transmitters allows enables precise tracking of individual movements and helps overcome the limitations of conventional visual observation (Reinert and Kodrich 1982; Weatherhead and Charland 1985; Ellis‐Soto et al. 2025). Nonetheless, transmitter mass is generally restricted to 5%–10% of an animal's body weight (Lutterschmidt 1994; O'Mara et al. 2014; Snijders et al. 2017), limiting its applicability to small‐bodied species such as lizards and particularly constraining its use in limbless taxa, including snakes.

In recent years, ethical considerations regarding animal welfare have assumed an indispensable role not only within the scientific community but also across all disciplines that involve the handling or study of animals (Speed et al. 2007; Perry et al. 2011). In response to these ethical constraints and the increasing prominence of bioethical debates, non‐invasive identification techniques based on individual body pattern characteristics have emerged as promising alternatives to traditional methods such as tissue clipping and tagging (Soulsbury et al. 2020; Klabukov et al. 2023). Moreover, recent studies have highlighted the potential of photographic and other visual datasets as effective and reliable tools for individual recognition (Miele et al. 2021; de Lorm et al. 2023). In reptilian taxa, including snakes and lizards, individual identification is widely achieved through the photographic documentation of scale pattern on specific anatomical regions such as the head, thorax, ear area, and jaw (Sacchi et al. 2010; Gardiner et al. 2014). The efficacy of this non‐invasive approach has been demonstrated through direct comparative assessments with conventional invasive techniques, including toe clipping (Perera and Perez‐Mellado 2004), and its reliability has been consistently demonstrated across multiple species (Mettouris et al. 2016). For instance, a study on the California whipsnake ( *Masticophis lateralis* ) successfully identified individuals by photographing the spotted patterns on the underside of the jaw, thereby demonstrating that these markings remained stable despite minor variations in body size and coloration (Alvarez and Murphy 2022). Similarly, a long‐term individual marking study of the European adder ( *Vipera berus* ) that combined genetic data with counts, shapes, and arrangements of head scales tracked individuals over 2–3 year intervals for up to 12 year. This study confirmed the stability of head scale patterns throughout the observation period (Bauwens et al. 2018). Therefore, photographic identification methods represent an effective alternative that minimize impact on animals while providing high reliability and long‐term consistency.

The Mongolian racerunner (*Eremias argus*) was first described in 1869 from a specimen collected in Mongolia. This small lacertid species, with has an average lifespan of less than five years and is distributed throughout northeastern Asia, is characterized by several distinct longitudinal series of dark dorsal spots extending from the head to the tail (Peters 1869; Kim et al. 2010; Lee et al. 2023). The Mongolian racerunner predominantly inhabits sandy habitats associated with coastal and riparian zones. However, these habitats have undergone a rapid decline due to extensive anthropogenic development along shorelines and riverbanks in recent decades (Cho and Son 2024). In response, the species has been designated as an Endangered Wildlife Species, Category II by the Ministry of Climate, Energy and Environment (MCEE) of the Republic of Korea to ensure its legal protection and systematic conservation management (NIBR 2019). In particular, the Korea National Park Service designated approximately 16,000 m^2^ of dune habitat within Taean Coast National Park, which is known encompass the largest population, as a Special Protection Zone in 2009 (Song et al. 2013; Chang et al. 2021). Previous studies have investigated population size estimates (Chang et al. 2021) and home range characteristics (Song et al. 2013) of this species. As most previous studies have relied on invasive marking techniques, there is a critical need for the development and application of non‐invasive identification methods to enable ethically sound and methodologically robust.

This study aimed to develop a minimally invasive individual identification technique that minimizes potential harm to target animals in population research. Specifically, we established a coding system based on dorsal spot patterns, assessed its accuracy, reliability, and field applicability for long‐term ecological monitoring. In particular, we focused on a photographic approach based on body pattern, which has been increasingly adopted in recent studies, highlighting its practicality and applicability under field conditions. Moreover, the method was designed to be accessible not only to professional researchers but also to citizen scientists, thereby facilitating broader participation in wildlife monitoring and conservation efforts. Ultimately, this approach is expected to serve as a feasible and reliable minimally invasive tool for population studies in wildlife research, particularly for *E. argus* .

## 2 Materials and Methods

### 2.1 Study Site and Photographic Data Collection

**FIGURE 1:**
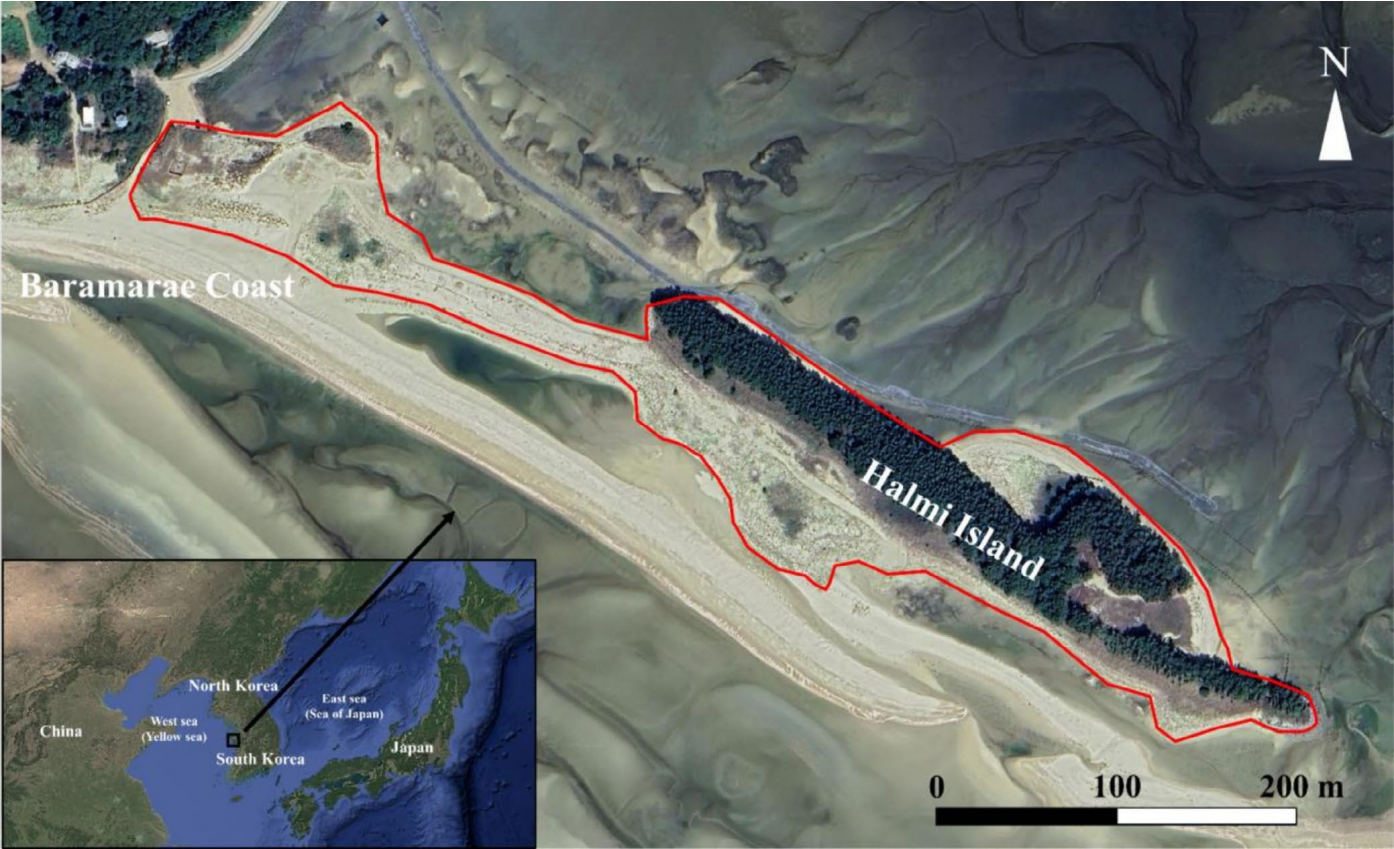
Baramarae Coast and Halmi Island, Taean‐gun, Chungcheongnam‐do—study site for individual identification and population size estimation of the Mongolian racerunner (*Eremias argus*). Red line denotes field survey range.

The study was conducted at Baramarae Coast, Gonam‐myeon, Taean‐gun, Chungcheongnam‐do, South Korea (N 36.411712, E 126.379408) (Chang et al. 2021). The total area of the study site was approximately 31,286 m^2^, encompassing an elongated island locally known as Halmi Island (Figure 1). Field surveys were conducted on May 17, June 15, and August 28, 2024. Individuals were captured by three researchers who conducted visual encounter surveys on foot within the study area (Chang et al. 2021). Dorsal photographs were taken of all captured individuals, and the coordinates of the initial capture locations were extracted from the GPS data embedded in the camera's metadata (Galaxy S23 plus, Samsung, South Korea). The collected coordinates were visualized on Google Earth, where positional errors were corrected by comparing them with the actual observed field locations on the map. To minimize handling stress, no morphological measurements or physical markings were performed. Each individuals was released at the exact point of capture immediately after photographic documentation.

### 2.2 Coding Procedure for Individual Identification

**FIGURE 2:**
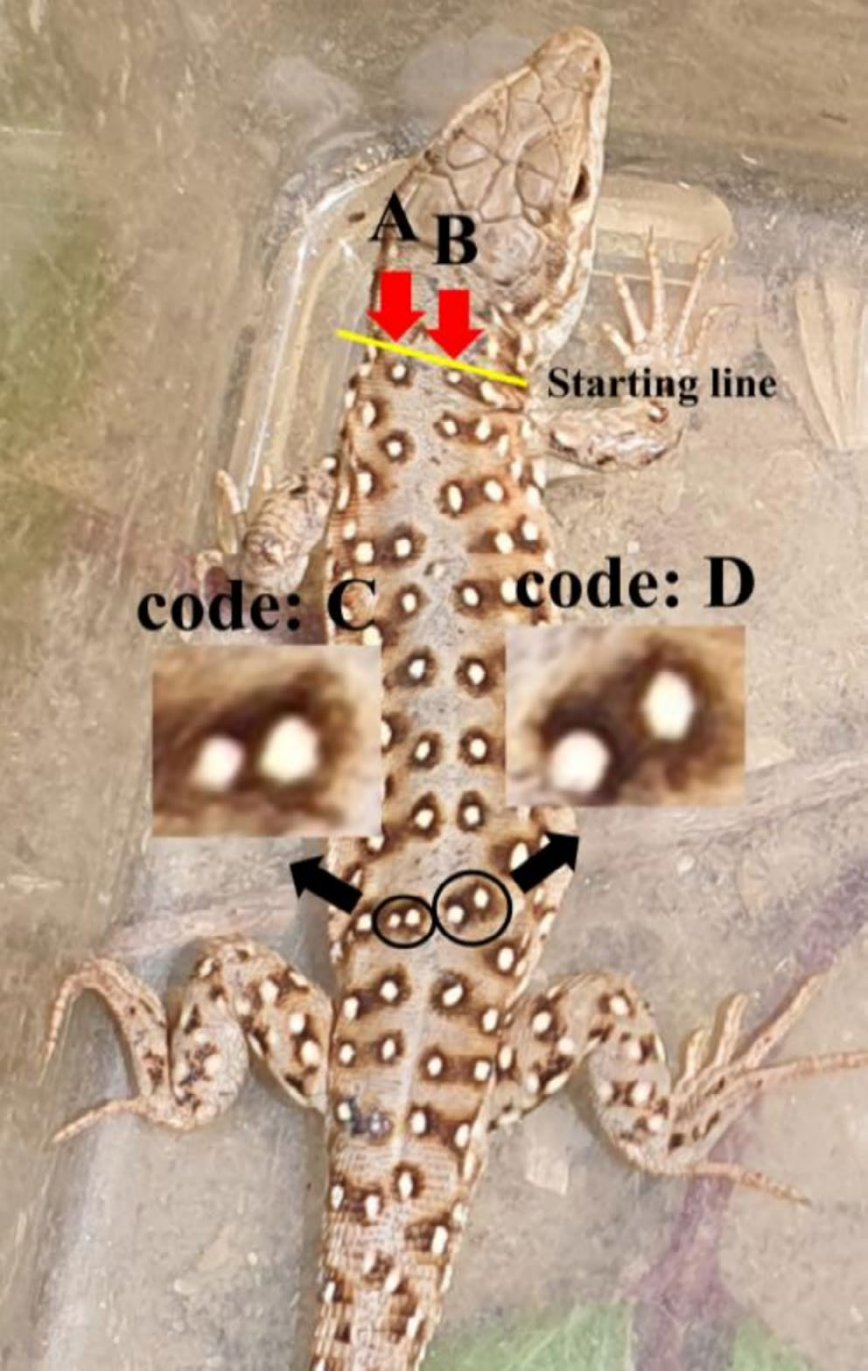
Example of individual marking in the endangered Mongolian racerunner (*Eremias argus*) using two central dorsal spot rows (indicated by red arrows). Regular spots on the left side of the body were labeled as “A,” overlapping spots on the left side as “C,” regular spots on the right side as “B,” and overlapping spots on the right side as “D.” The code of this individual is “BABABAABABBABADCBAABBABAAB”.

**FIGURE 3:**
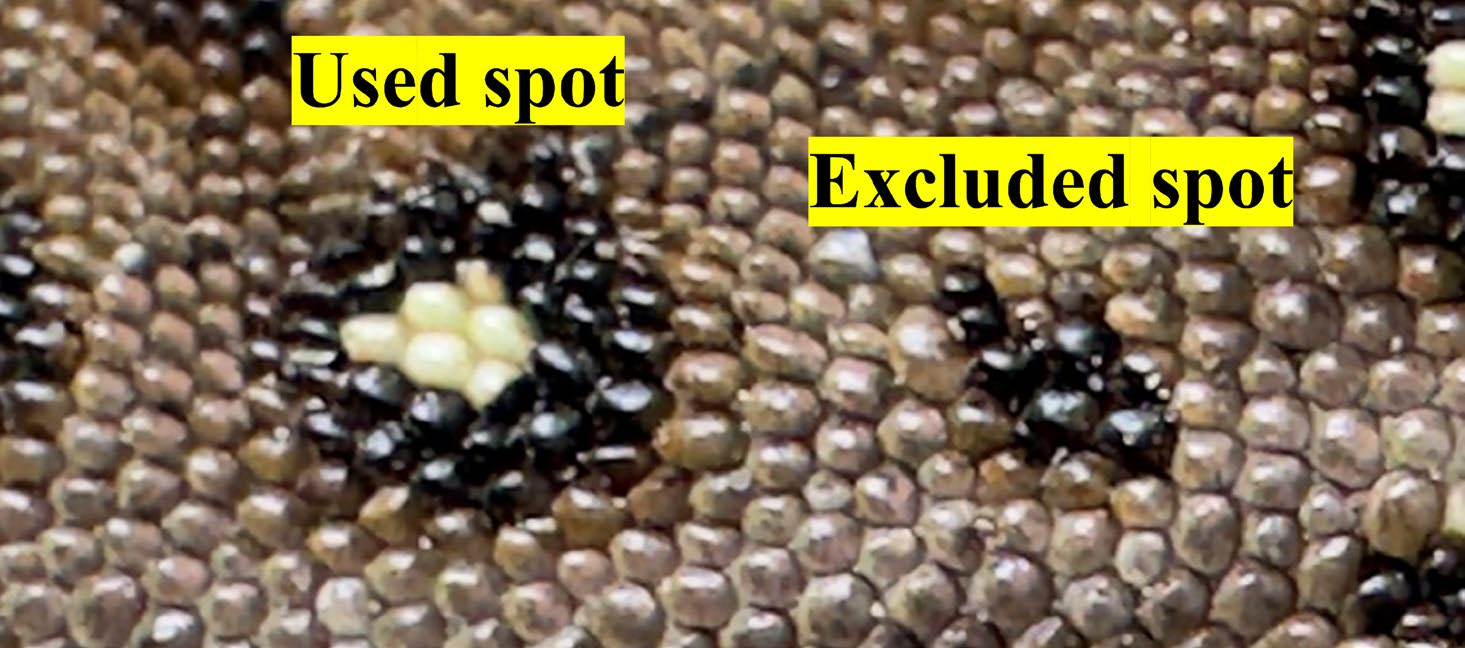
Classification of dorsal spots on the body surface. Spots with well‐defined, white centers surrounded by dark area (“Used spot,” left) were included in the analysis, whereas small and irregular spots (“Excluded spot,” right) were omitted.

Each individual was assigned a unique code based on the position and morphology of the dorsal spots. The following five coding rules were applied. First, two longitudinal rows of spots on the left and right sides of the body midline were used for coding (Figure 2). This region was selected because it exhibited consistent spot patterns among individuals. Second, spots were numbered sequentially from head to tail. Third, to distinguish the left and right sides, spots on the left row were labeled “A,” and those on the right row were labeled “B” (Figure 2). Fourth, if a spot overlapped with adjacent spot, an extra code was added. Overlapping spots on the left were labeled “C,” and those on the right were labeled “D” (Figure 2). Fifth, only well‐defined spots were included for coding, specifically those in which the dark margin covered more than 50% of the white area, while smaller or poorly defined spots were excluded (Figure 3). Using these criteria, the individual shown in Figure 2 was assigned the code “BABABAABABBABADCBAABBABAAB.” All assigned codes were recorded in Microsoft Excel.

### 2.3 Capture and Research Authorization

*Eremias argus* is designated and protected as a Category II Endangered Wildlife Species by the Ministry of Climate, Energy and Environment (MCEE). Individual identification methods were implemented on individuals captured under permits and research approval from the Geum River Basin Environmental Office (Permit No.: ED202405ECP0016). The study site, Baramarae Coast, was identified as the largest known population of *Eremias argus* in South Korea in earlier studies conducted in 2005. Based on these findings, the Korea National Park Service designated this area as a Special Protection Zone for *E. argus* in accordance with the National Park Service Act. In accordance with the Korea National Park Service Act, permits for the management and research of special protection zones and protected species were obtained from the Korea National Park Service on April 29, 2024 (KNPRI20242128).

### 2.4 Individual Identification and Analysis Using Spot Patterns

We developed and applied the individual identification method to all captured individuals (Appendix S1). The sorting function in Microsoft Excel was then used to identify duplicate individuals and detect recaptures. Subsequently, the frequency and characteristics of overlapping dorsal spots identified during the coding process were analyzed. The individual identification method was assessed using descriptive summaries (prevalence and frequency of overlapping spots) and inferential tests (differences in the number of overlapping spots between left and right sides; code length differences). These comparisons were evaluated using the non‐parametric Wilcoxon signed‐ranks test, with statistical significance set at 0.05. All statistical values were expressed as mean (± SD).

## 3 Results

Across the field survey, 72 individuals were captured on May 17, 61 on June 15, and 42 on August 28, yielding 175 capture events across 166 unique individuals (Table 1).

**TABLE 1:** Summary of capture events and individual records across survey sessions.

Survey date	Total captures	Newly captured individuals	Recaptured individuals	Cumulative unique individuals
17‐May	72	72	0	72
15‐Jun	61	56	5	128
28‐Aug	42	38	4	166

The mean code length per individual was 25.9 ± 3.0 (*n* = 166, range: 19–36). When using overlap codes C and D, the mean code length decreased to 25.0 ± 2.5 (*n* = 166, range: 19–32). Code counts differed significantly depending on overlap code usage (Wilcoxon signed‐rank test *V* = 3321.00, SE = 207.543, *z* = 8.001, *p* < 0.001, *n* = 166).

When only the basic codes A and B were used for individual identification, an average of 12.5 ± 4.8 codes per individuals was required (*n* = 162, range = 1–25). In contrast, when overlapping spot codes C and D were included, individuals were identified using an average of 11.6 ± 4.9 codes (*n* = 162, range = 1–21). The required codes length was significantly lower when overlapping spot codes were utilized (Wilcoxon signed‐ranks test *V* = 4391.00, SE = 521.461, z = −2.152, *p* = 0.031, *n* = 159).

Among the 166 individuals, 48.8% (81 individuals) showed code C or D at least once. The mean number of overlapping spots per individual was 1.8 ± 1.3 (*n* = 81), with a maximum of 10 within a single individual. On the left side (code C), 60 individuals showed an average of 1.4 ± 0.8 overlappping spots (maximum = 5), while 51 individuals on the right side (coded D) showed an average of 1.2 ± 0.7 overlappping spots (maximum = 5). The frequency of overlapping spots was significantly higher on the left side than on the right side (Wilcoxon signed‐ranks test *V* = 3.00, SE = 9.552, z = −2.565, *p* = 0.010, *n* = 30).

**FIGURE 4:**
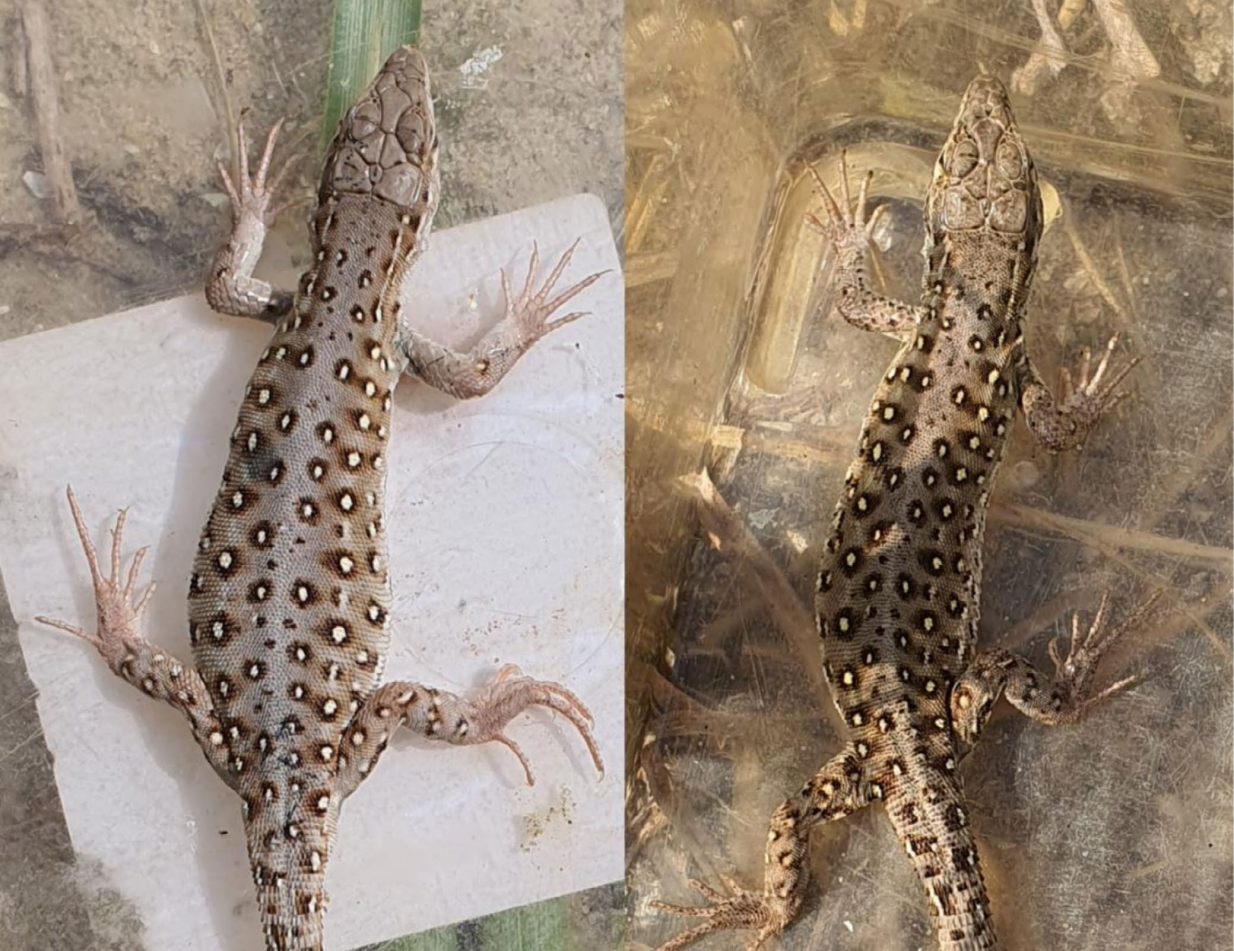
A female (left) captured on May 17, 2024, and the same female (right) recaptured on August 28 2024. Eggs were observed in the abdomen at the first capture, while at recapture, oviposition had already been completed. The black bruise on the left side of the body and the wound near the tail (caused by the male's bite during the breeding process) were observed at the first capture but had disappeared by the time of recapture. In contrast, no changes were observed in the dorsal spot patterns and numbers.

A total of 9 recapture events were recorded during the field surveys (Table 1). The recapture rate was 8.2% in the second survey (*n* = 5/61) and 9.5% in the third survey (*n* = 4/42). No positional changes in dorsal spot patterns were detected across recaptures (Figure 4). These findings indicate that overlapping spot codes improve identification efficiency without compromising long‐term stability.

## 4 Discussion

In this study, we proposed and applied—for the first time—a method to code and identify individual Mongolian racerunner (*Eremias argus*) based on their unique dorsal patterns for population monitoring. This approach enabled reliable individual recapture identification and demonstrates strong potential for use in minimally invasive or non‐invasive population size estimation studies. However, while dorsal spot pattern stability has been confirmed in other species, this has not been specifically verified in Mongolian racerunners. For long‐term monitoring applications, additional studies confirming that dorsal spot patterns remain stable over time in this species are required.

### 4.1 Minimally Invasive Individual Identification Using Spot Patterns

The spot pattern‐based individual identification method proposed in this study offers several practical advantages. The foremost consideration in developing this approach was its minimally invasive applicability for ecological research. Although the capture of individuals is necessary to obtain dorsal photographs, no additional manipulations are performed, thereby reducing stress on the animals.

The second advantage of this method is that it does not require any specialized equipment for individual identification. Since the method only requires dorsal photographs, the necessary data can be obtained using a standard camera or even a smartphone. In contrast, tagging‐based identification studies require additional devices and procedures, such as chip implantation and the use of specialized equipment, such as needles, and readers for recognition (Penney et al. 2001; Gibbons and Andrews 2004; Recio et al. 2019). Although previous image‐based identification methods have utilized recognition programs such as I3S Pattern (Rocha et al. 2013; Gardiner et al. 2014), our approach encodes the dorsal spot patterns of each individual, making identification straightforward and data management highly efficient. Moreover, even the widely used Excel program is sufficient to verify recapture events. If a simple mobile application were developed, the entire identification process could be performed on a single smartphone.

The third, because the method utilizes variation in spot patterns, the generated individual codes inherently reflect the unique characteristics of each animal. This individuality minimizes the likelihood of code duplication as the number of sampled individuals increases and can serve as a clear indicator for more accurate identification.

The fourth and possibly most important advantage is the method's simplicity and rapid application. Each code requires only four elements (A–D), including intersection points, and in some cases, accurate coding is possible with only two elements (A, B). Furthermore, due to the simplicity of the coding process, once trained, a researcher can assign a code to an individual within 10 s. These attributes reinforce the method's field applicability and low‐invasiveness, making it an effective tool for future use by field researchers and citizen scientists alike.

### 4.2 Application and Extension of the New Minimally Invasive Approach

Although the method proposed in this study is minimally invasive and rapid, it also has some several limitations. For example, the target species must (1) possess distinct spot patterns (or other distinguishable markings), (2) exhibit minimal changes in those patterns over time, and (3) display a relatively stable and consistent pattern that can be coded. Conversely, this implies that the proposed method can be applied not only to lizards but also to any species across various taxonomic groups that exhibit similar distinctive patterns. Furthermore, the most distinctive feature of this approach is that it encodes individual‐level variation. For instance, Gardiner et al. (2014) identified Eastern Water Dragons using scales surrounding the tympanum. In our framework, the morphological traits employed in that study—such as variations in scale size (smaller or larger), shape, or arrangement—could be systematically encoded. If features such as relative scale size, color frequency (e.g., ratios of white, yellow, and black pigmentation), shape (circular or oval), and arrangement were coded, individual identification would be feasible solely through photographic data, without the need for specialized equipment (Speed et al. 2007; Schoen et al. 2015; Tzika et al. 2023, 2024). These findings suggest that the coding framework developed in this study can be extended to pattern‐based individual identification in a wide range of species. Future studies could integrate this coding framework with automated image‐recognition algorithms or mobile applications or web‐based platforms, enabling faster and more scalable identification processes in large population studies.

### 4.3 Overlap of Spot Patterns and Lateral Asymmetry

Asymmetry in the number, shape, or arrangement of body markings is commonly observed across various animal taxa, and its causes and biological significance are influenced by multiple factors (Brown et al. 2017; Castillo and González‐Rivas 2023). For instance, in the Spotted salamander ( *Ambystoma maculatum* ), differences in the number of spots between the left and right sides have been shown to correlate with asymmetry in limb length (Davis and Maerz 2007). Such asymmetry is often shaped by developmental stress and may serve as an indicator of stress sensitivity. Additionally, asymmetrical patterns can have ecological functions, such as enhancing the effectiveness of camouflage or reinforcing visual communication signals (Langridge 2006). In the Trinidadian guppy ( *Poecilia reticulata* ), females have been shown to prefer males with asymmetric coloration patterns (Sheridan and Pomiankowski 1997a). Numerous studies have thus suggested that asymmetry is not merely an incidental developmental error but rather reflects evolutionary pressures, sexual selection, and adaptive strategies that related to survival (Sheridan and Pomiankowski 1997b; Wood 1997). Although the present study does not address asymmetry in detail, future investigations examining the frequency of asymmetry in relation to sexual dimorphism or survival rates may provide valuable insights into the biological significance of dorsal spot pattern asymmetry in the Mongolian racerunner.

## 5 Conclusion

The spot pattern‐coding method proposed in this study demonstrated the ability to accurately identify individuals using simple photographic records. This approach is expected to minimize stress and physical harm to animals during research activities. Unlike conventional tagging techniques, it allows individual monitoring without specialized equipment, making it suitable for field‐based studies and citizen science programs. Although its applicability is limited to individuals with distinct and stable body markings, it can be extended to a wide range of taxa when such conditions are met. Nevertheless, such limitation also defines the specificity of the method. Moreover, integration with mobile applications could further enhance its accessibility and efficiency in the field. Ultimately, this method represents a novel, minimally invasive, and welfare‐oriented approach that may serve as a new standard for individual identification in population monitoring and endangered species conservation.

## Author Contributions

**Jae Young Song:** conceptualization (equal), formal analysis (supporting), funding acquisition (lead), investigation (supporting), methodology (supporting), writing – original draft (supporting). **Min Ho Chang:** data curation (supporting), investigation (supporting), writing – review and editing (supporting). **Seok Beom Kim:** data curation (supporting), investigation (supporting), writing – review and editing (supporting). **Kyo Soung Koo:** conceptualization (equal), formal analysis (lead), investigation (equal), methodology (lead), project administration (lead), supervision (lead), visualization (lead), writing – original draft (lead).

## Conflicts of Interest

The authors declare no conflicts of interest.

## Supporting information


**Appendix S1:** ece373438‐sup‐0001‐Appendix1.xlsx.

## Data Availability

All the required data are uploaded as Supporting Information.
